# Knowledge, attitudes, and practices towards the use of GLP-1 receptor agonists for weight loss among the general population in Jordan; A cross-sectional study

**DOI:** 10.1371/journal.pone.0314407

**Published:** 2024-12-05

**Authors:** Rana Abutaima, Muna Barakat, Hana M. Sawan, Shatha M. Al Omari, Nizar M. Mhaidat

**Affiliations:** 1 Faculty of Pharmacy, Zarqa University, Zarqa, Jordan; 2 Faculty of Pharmacy, Applied Sciences Private University, Amman, Jordan; 3 Department of Clinical Pharmacy, Faculty of Pharmacy, Jordan University of Science and Technology, Irbid, Jordan; University of Petra (UOP), JORDAN

## Abstract

**Introduction:**

Obesity has emerged as a global pandemic, with its prevalence notably increasing during the COVID-19 lockdown of 2019. In response, many individuals have turned to pharmacological interventions, including antidiabetic medications, as means of achieving weight loss with minimal effort. This study aims to assess the knowledge, attitudes, and practices of the Jordanian population regarding the use of antidiabetic agents, specifically glucagon-like peptide-1 receptor agonists and biguanides, for weight management.

**Method:**

A self-administered validated online questionnaire was developed and disseminated to public utilizing a cross-sectional design. Data were extracted to examine descriptive statistics. Linear regression was performed to evaluate associations with knowledge and attitude. A *p*-value ≤0.05 was chosen to indicate statistical significance.

**Results:**

Total of 389 responses were analyzed, 65.6% females, 54.2% married, 78.1% living in the center of Jordan, 35.5% overweight and 26.5% have >1000 Jordanian Dinars monthly income. Fifty seven percent of the study participants think that antidiabetics could be used for weight loss. 47.27%, 44.55%, 68.18% recognized glucagon-like peptide-1receptor agonists; (Ozempic^®^), (Saxenda^®^), (Mounjaro^®^) as well as (Glucophage^®^) use for weight loss, respectively and 12.3% of participants used medications to lose weight. Neutral attitude was observed. Gender and body mass index were significantly affecting the participants knowledge (*p*<0.001, *p* = 0.002, respectively).

**Conclusion:**

Use of (Ozempic^®^), (Saxenda^®^) and other antidiabetics to lose weight become a common practice. The results of this study suggests supervising prescription and dispensing to avoid misuse, especially, in people who are contraindicated to use them.

## Introduction

According to the World Health Organization (WHO), obesity is defined as having a body mass index (BMI) greater than 30 kg/m^2^ [1]. However, this definition differs from one nation to another based on the fat distribution which is considered a major factor for predicting comorbidities [2]. Obesity is recognized as a risk factor for many chronic diseases such as hypertension, diabetes mellitus type II and other coronary heart disease [3, 4]. Consequently, managing body weight within a BMI value of 18–25 kg/m^2^ as well as avoiding fat accumulation in the viscera beyond 15% and 25% in males and females, respectively is a health necessity [5].

A national survey in Jordan was conducted in 2017 by *Ajlouni et al* to investigate the rate of obesity among both males and females and the risks of developing comorbidities [6]. The findings revealed that the male and female odd ratios (OR) for obesity are 1.98 (n = 1193) and 1.96 (n = 2863), respectively, which are twice as high as they were in the 2009 evaluation [6]. Additionally, risk of type II diabetes, hypertension and high triglycerides level has increased [6]. The findings of this study were adopted by the WHO and documented a percentage prevalence of obesity of 60.4 and 75.6 in men and women, respectively [7]. Similarly, The United nations Global Health Targets report for 2025 stated that Jordan has one of the highest percentages of obesity in both genders and the chances to meet obesity targets by 2025 are considered poor ≤ 1% [8].

Recent evidences suggest that people started to consider convenient tools and/or methodologies for weight reduction or management such as bariatric surgeries, weight loss medications and more recently are injectable hypoglycemic agents [9, 10]. For instance, the glucagon-like peptide-1 (GLP-1) receptor agonists; Semaglutide, (Ozempic^®^), Liraglutide, (Saxenda^®^) and Tirzepatide, (Mounjaro^®^) [11–13]. (Ozempic^®^), (Mounjaro^®^) and the biguanide agent; metformin (Glucophage^®^) are hypoglycemic agents recommended in treating patients with type II diabetes [14–16]. The use of these agents for weight loss is considered off-label, with some not approved for this indication by the Food and Drug Administration (FDA) [11, 15, 16]. On the contrary, a higher dose of Semaglutide that is licensed under the name (Wegovy^®^) as well as (Saxenda^®^) are FDA-approved for weight loss [17–19]. Additionally, American Diabetes Association Standards of Medical Care in Diabetes recommends using GLP-1 receptor agonists with high to very high glucose and weight lowering efficacy such as Tirzepatide or Semaglutide or liraglutide in patients with type II diabetes who need a weight management plan [20]. On the other hand, the Endocrine Society is conservative about the use of antidiabetics for weight loss for the same aforementioned group [21]. Whereas the European Association for the Study of Obesity released a position statement regarding the use of antidiabetics for weight loss [22]. Collectively, large dose of Semaglutide (*i*.*e*. Wegovy^®^) or (Saxenda^®^) alongside adapting healthy lifestyle to promote weight loss were recommended. Nevertheless, previous studies have reported adverse events development such as gastrointestinal, kidney disease, pancreatitis, gallbladder problems and most importantly, thyroid tumors and cancer upon using certain antidiabetic agents for weight loss [23–25].

In developing countries, access to prescription medications such as (Ozempic^®^), (Saxenda^®^), (Mounjaro^®^) and (Glucophage^®^) without medical prescription is possible and increased with the possibility of buying them over the internet [26, 27]. For instance, social media platforms (*i*.*e*. Facebook^®^, Instagram^®^, Twitter^®^, Whatsapp^®^) have changed the buying behaviors of their users, relying mostly on other users’ experience rather than healthcare providers’ recommendations [28]. One of these buying and using behaviors are (Ozempic^®^) and (Saxenda^®^) use for weight loss [11, 29]. This might result in improper use, especially in people with risk factors to develop cancer, kidney disease or low blood glucose levels. However, the fact that users of these injectable medications rely on peers experience or reviews from other users on social media platforms impose more health risks, specifically adverse events and long-term effects that are not well-understood yet. In Jordan, self-medication and relying on non-scientific resources to gain knowledge about pharmacotherapeutic agents are not uncommon [30, 31]. Considering that there is no national or regional knowledge, attitudes and practices (KAP) assessment for the use of antidiabetics for weight loss purposes, this study was designed to assess the Jordanian population KAP and buying behaviors of (Ozempic^®^), (Saxenda^®^) and (Mounjaro^®^) for the purposes of weight loss. A secondary objective was to explore the factors affecting the knowledge and attitude toward antidiabetics use for weight loss among the study target group.

## Methodology

### Study design and setting

This study was an observational cross-sectional study. It was conducted using a self-administered online questionnaire that is accessible *via* both smartphones and computers. The questionnaire was disseminated using different social media platforms such as Facebook^®^, Instagram^®^, Twitter^®^, Whatsapp^®^ to collect individual responses of the target group over the period 4^th^ to 25^th^ of August 2023. Participants were invited to fill out the questionnaire *via* national social media groups, especially it was observed there is a huge debate over the summer period about the uses of antidiabetic agents for weight loss. Potential bias in data collection was effectively mitigated by configuring the questionnaire to restrict responses to a single submission per participant, thereby ensuring that each individual could only provide one response.

### Ethical approval

This study was granted ethical approval after protocol review by the institutional review board (IRB) committee at Applied Sciences University with an approval number (2023-PHA-29) on 10^th^ of July 2023. Confidentiality and anonymity of data were reassured and this was identified to participants prior to responses collection in the provided written informed consent before taking part in the study. Respondents were informed that they can withdraw from participation at any time during questionnaire filling out and the data they shared will solely be used for research purposes.

### Participants and sample size

An estimated sample size of 385 was calculated with a margin of error of 0.05 and 95% confidence interval and according to the total number of Jordanian population using *Raosoft Sample Size online calculator* [32]. Any adult living in Jordan was eligible to fill-out this questionnaire.

### Questionnaire content development and validation

A self-administered survey was developed using Google^®^ forms after reviewing the literature [33]. The questionnaire consists of four main sections. The first one addresses participants demographics such as age, gender, marital status, weight, height, education, family monthly income, place of residence, being health insured and having chronic diseases or not. Weight and height were utilized to calculate the BMI (kg/m^2^) that was then categorized according to the WHO scale. The second section evaluated participants’ knowledge of antidiabetic medications in five main questions. For instance, participants were asked about the possibility of using certain antidiabetic medications for weight loss (*e*.*g*. (Ozempic^®^), (Saxenda^®^) and (Mounjaro^®^), (Glucophage^®^), Insulin, glimepiride (Amaryl^®^) and empagliflozin (Jardiance^®^) and approval by regulatory agencies for this indication (S1 Appendix). The third section evaluated participants’ attitudes within the society regarding the use of antidiabetic medications for weight loss purposes. Based on a five degree Likert scale of strongly agree, agree, neutral, disagree and strongly disagree, participants were requested to provide their opinion about level of regulatory authorities supervision on dispensing of antidiabetic medication for weight loss, as well as using these medications under medical supervision (S1 Appendix). The last section evaluated the practices of Jordanian population in using injectable antidiabetic agents for weight loss *i*.*e*. GLP-1 receptor agonists in addition to metformin (Glucophage^®^). Only participants who used antidiabetic agents for weight loss proceeded to this section.

Participants were requested to declare which antidiabetic agent they have used from the following medications (Ozempic^®^), (Saxenda^®^) and (Mounjaro^®^) and (Glucophage^®^). Other common practices to lose weight were also evaluated. Data regarding checking the source of antidiabetic medications, difficulty in obtaining them, the lost weight (if applicable), method of verifying the medication sources, doctors’ consultation, weight gain after medication cessation, adverse events development and reading medical information prior to use were collected in this section. Participants were also offered the option if they would like to share more information about their experience in using antidiabetic agents for weight loss. Pictures of the brand names of study medications were provided within the questionnaire to help people identify them. All questions that were included in the study questionnaire are provided in S1 Appendix.

### Questionnaire validation

The validity of the questionnaire was ensured through face and content validation, where a group of experts and lay participants reviewed the questionnaire to confirm that it accurately covered all aspects of the topic. Reliability was assessed using Cronbach’s alpha, which demonstrated acceptable internal consistency, confirming that the questionnaire’s scales consistently measured the intended constructs.

Therefore, face and content validity was assessed after piloting the questionnaire to 12 subjects (5 academics and 7 from the public). As the academic participants have experience in the field of the study and were capable to check the construction of the questionnaire and the content validity that fully cover the topic. In addition, the pilot participants’ feedback which was related to any scientific jargon or unclear questions were taken into account and the questionnaire was updated accordingly where applicable. Most of the feedback received from the pilot sample focused on the knowledge section. Examples of the feedback received were: types of chronic diseases (*e*.*g*. obesity). A question was added to the knowledge section if antidiabetic agents could be used for weight loss purposes. Other questions were removed such as the types of antidiabetic agents used in treatment of polycystic ovaries. Adverse events of antidiabetic agents were summed up in one question instead of two. The questionnaire was first designed in Arabic; the national language of Jordan and was translated to English by two academics who are fluent in both languages. Responses were collected in Arabic which is the national and spoken language in Jordan.

Regarding the reliability, Cronbach’s alpha of the questionnaire was 0.81, *i*.*e*., that the scales built were appropriate for the task at hand. Values above 0.7 indicate acceptable internal consistency.

### Scoring system and statistical analysis

Knowledge was assessed by giving 1 to the correct Answer and 0 to the Wrong Answer. The scale measured knowledge from a maximum of 27 to a minimum of Zero. The score <5.4 were considered very unconfident, 5.4–10.8 as fairly unconfident, 10.9–16.2 as neutral, 16.3–21.6 as fairly confident, 21.7–27 as very confident knowledge about weight reduction medication. Attitude score was assessed using Likert scale with a maximum of 5 to a minimum of 1. Scores were categorized using Bloom’s cut-off points: < 3 (<59%) were considered negative attitude, 3–3.9 (60.0–79.0%) as neutral and 4–5 (80.0–100.0%) as a positive attitude toward weight reduction medication [34]. Bloom’s cut-off points are considered one of the standard categorizing tools for KAP studies following calculation of their scores [34]. As it helps to understand and analyze the scores outcomes, and then reveals the recommendations and conclusion of the study [34].

Data were exported from Google^®^ Forms into an Excel^®^ spreadsheet, entered into the Statistical Package for Social Sciences version 24.0 (SPSS^®^ Inc., Chicago, IL, USA), and statistically analyzed. The mean and standard deviation (SD) for continuous variables were employed in descriptive statistical analysis to analyze the sociodemographic data. While categorical variables were presented as frequencies and percentages. To evaluate the normalcy, the Shapiro-Wilk test was applied. No data were missing as all questions were mandatory to submit the form.

Using univariate linear regression analysis, independent factors that might influence participants’ knowledge and attitude regarding weight reduction medications were looked at. Then, variables that had been determined to be significant on a single factor level (P<0.25 in each case) were added to multiple linear regression models. After confirming their independence, variables were chosen; tolerance values > 0.1 and Variance Inflation Factor (VIF) values <10 were tested to show that multicollinearity between the independent variables in regression analysis was not present. No included variable had multicollinearity; as a result, none was deleted. Noteworthy, a VIF according to [35] higher than 10 or tolerance is lower than 0.1, there is significant multicollinearity that needs to be corrected. *p*-value ≤0.05 was used to determine statistical significance.

## Results

### Sociodemographic characteristics

A total of 395 responses were received by the participants; six responses were excluded due to reluctance to consent. Three hundred eighty-nine responses were included in the final analysis. More than half of the respondents were female (255, 65.6%), married (211, 54.2%), living in the central area of Jordan (304, 78.1%), and holding a bachelor’s degree (219, 56.3%). Most of the participants worked in the medical field (190, 48.8%) and had medical insurance (255, 65.5%). The BMI was normal for 152, 39.1% of participants, Table 1.

**Table 1:** Sociodemographic characteristics of the participants (n = 389).

Variable	n	%
• **Age (in years)**		
• <25	168	43.2
• 25–30	138	35.5
• >30	83	21.3
**Gender**		
• Male	134	34.4
• Female	255	65.6
**Marital status**		
• Single	170	43.7
• Married	211	54.2
• Others	8	2.1
**Residencial area**		
• North governorates (Irbid, Jerash, Ajloun, Mafraq)	62	15.9
• Central governorates (Amman, Zarqa, Balqa, Madaba)	304	78.1
• Southern governorates (Karak, Tafila, Ma’an, Aqaba)	23	5.9
**Monthly income of the family (Jordanian Dinars)**		
• Less than 300	70	18.0
• 300–700	145	37.3
• 701–1000	71	18.3
• More than 1000	103	26.5
**The highest degree or level of education you have completed**		
• High School	36	9.3
• Vocational education	1	0.3
• Diploma	40	10.3
• Bachelor’s degree	219	56.3
• Postgraduate degree (Master’s, Ph.D.)	93	23.9
**Occupation (profession)**		
• Medical occupations (medicine, pharmacy, nursing, laboratories…etc.)	190	48.8
• Non-medical occupations (engineering, information technology, arts, Craft occupations, etc.)	155	39.8
• Not applicable	44	11.3
**Health insurance status**		
• Insured	255	65.6
• Uninsured	134	34.4
**Presence of chronic diseases**		
Do you suffer from chronic diseases?		
• Yes	85	21.9
• No	304	78.1
**If yes, what are these diseases?** *		
• Diabetes	26	6.7
• Hypertension	30	7.7
• Cardiovascular disease	14	3.6
• Thyroid diseases	14	3.6
• Hyperlipidemia	31	8.0
• Obesity	35	9.0
• Others	29	7.5
**BMI Categories** ^$^		
• Severely Underweight (< 16.0)	0	0.0
• Underweight (16.0–18.4)	16	4.1
• Normal (18.5–24.9)	152	39.1
• Overweight (25.0–29.9)	138	35.5
• Moderately Obese (30.0–34.9)	53	13.6
• Severely Obese (35.0–39.9)	23	5.9
• Morbidly Obese (> 40.0)	7	1.8
	**Mean**	**SD**
**Height (cm)**	166.46	9.2
**Weight (kg)**	73.64	18.0

*More than one option was allowed,

^$^ according to the WHO categorization of BMI. No data were missing for the 389 individuals who were statistically analyzed.

### Participants’ knowledge about antidiabetic medications uses for weight reduction

**Fig 1:**
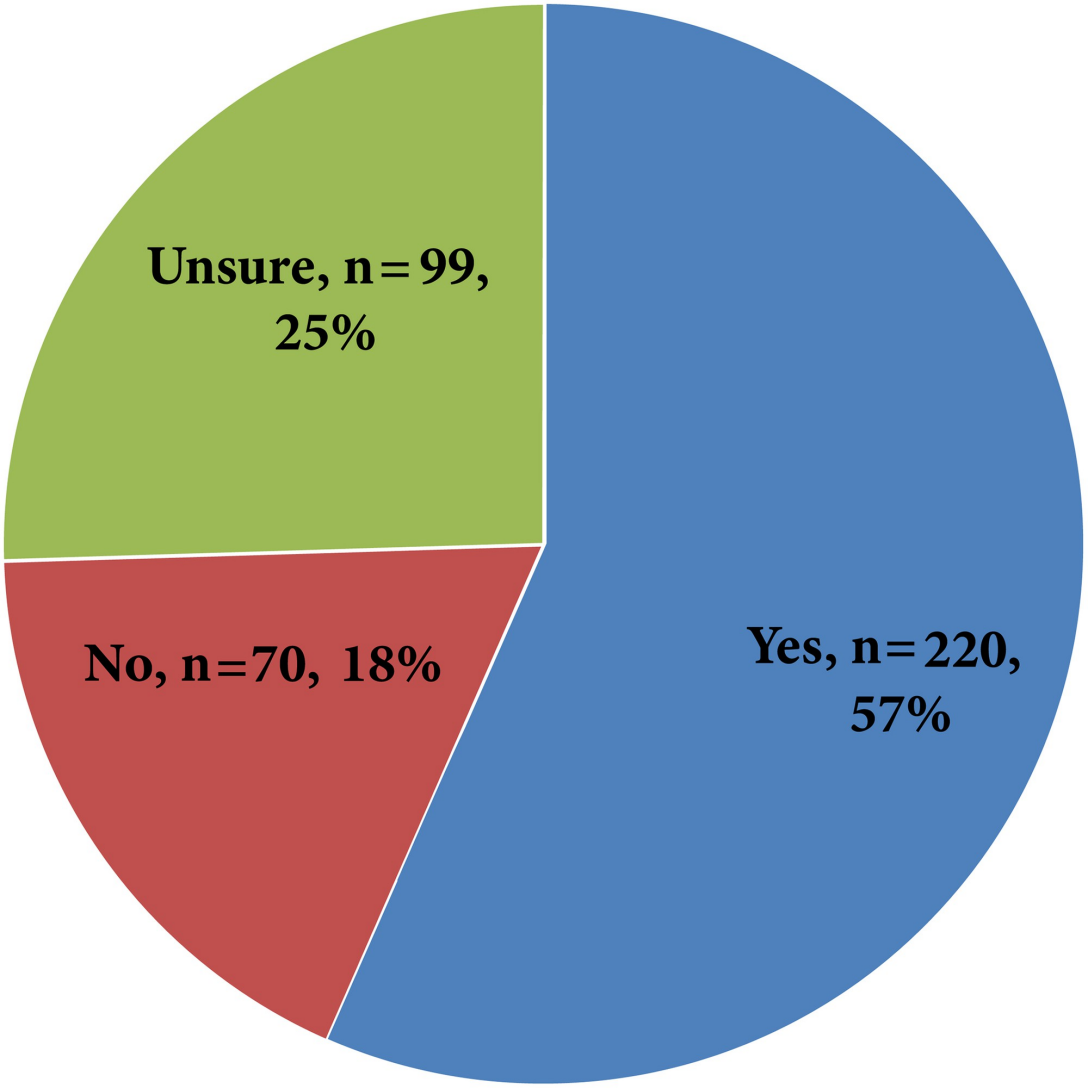
Participants’ responses regarding the possible use of some antidiabetic medications for weight reduction (n = 389).

Upon asking participants about the possible use of some antidiabetic medications for weight reduction, more than half of them agreed with their use (220, 57%), while 99 (25%) participants were unsure, Fig 1.

In general, the knowledge score about this medication showed that study participants were fairly unconfident, as the mean and SD were 6.2±3.03 (out of 27), Table 2. Among the participants who agreed with their use (n = 220), almost one-third of the participants chose (Glucophage^®^), as a possible medication used for weight loss (150, 68.2%). At the same time, 47.3% and 44.5% (n = 104, 98, respectively) of the respondents reported (Ozempic^®^) and (Saxenda^®^, FDA-approved for weight loss). Most of participants declared that (Ozempic^®^) and (Saxenda^®^) are not approved (143, 65% and 189, 85.91%, respectively). Regarding the knowledge about side effects of the injectable weight reduction medications, more than half stated nausea/vomiting (128, 58.2%), Fatigue (120, 54.6%), diarrhea (115, 52.3%) and headache (111, 50.5%), Table 2.

**Table 2:** Participants’ knowledge about weight reduction medications (n = 220). More than one option was allowed.

Questions	Correct answer	
	n	%
**To the best of your knowledge, which of the following medications can be used to lose weight?**		
• (Ozempic^®^)	104	47.27
• (Saxenda^®^)^$^	98	44.55
• (Mounjaro^®^)	47	21.36
• (Glucophage^®^)	150	68.18
• Insulin	93	42.27
• (Amaryl^®^)	88	40.00
• (Jardiance^®^)	64	29.09
**Which of the following medications is approved to be used for weight loss by regulatory authorities such as the Food and Drug Administration?**		
• (Ozempic^®^)	143	65.00
• (Saxenda^®^) ^$^	76	34.55
• (Mounjaro^®^)	189	85.91
• (Glucophage^®^)	85	38.64
• Insulin	82	37.27
• (Amaryl^®^)	74	33.64
• (Jardiance^®^)	66	30.00
**To the best of your knowledge, what are the side effects of the injectable medications used for weight loss?**		
• Nausea and vomiting	128	58.18
• Diarrhea	115	52.27
• Abdominal pain	111	50.45
• Headache	105	47.73
• Fatigue	120	54.55
• Weight gain	108	49.09
• Skin swelling or irritation where the needle was inserted	72	32.73
• Dark patches of skin	49	22.27
• Unwanted sexual responses (sexual dysfunction or arousal)	34	15.45
• Depression	63	28.64
• Pancreatitis	53	24.09
• Tumors such as thyroid tumors	47	21.36
• Urinary tract infection	49	22.27
**Total knowledge score (out of 27)** *	**Mean**	**SD**
	6.2	3.03

*Knowledge was assessed by giving 1 to the correct Answer and 0 to the Wrong Answer. The scale measured knowledge from a maximum of 27 to a minimum of Zero. The score <5.4 were considered very unconfident, 5.4–10.8 as fairly unconfident, 10.9–16.2 as neutral, 16.3–21.6 as fairly confident, 21.7–27 as very confident knowledge about weight reduction medication.

^$^ FDA approved medication for weight loss.

**Fig 2:**
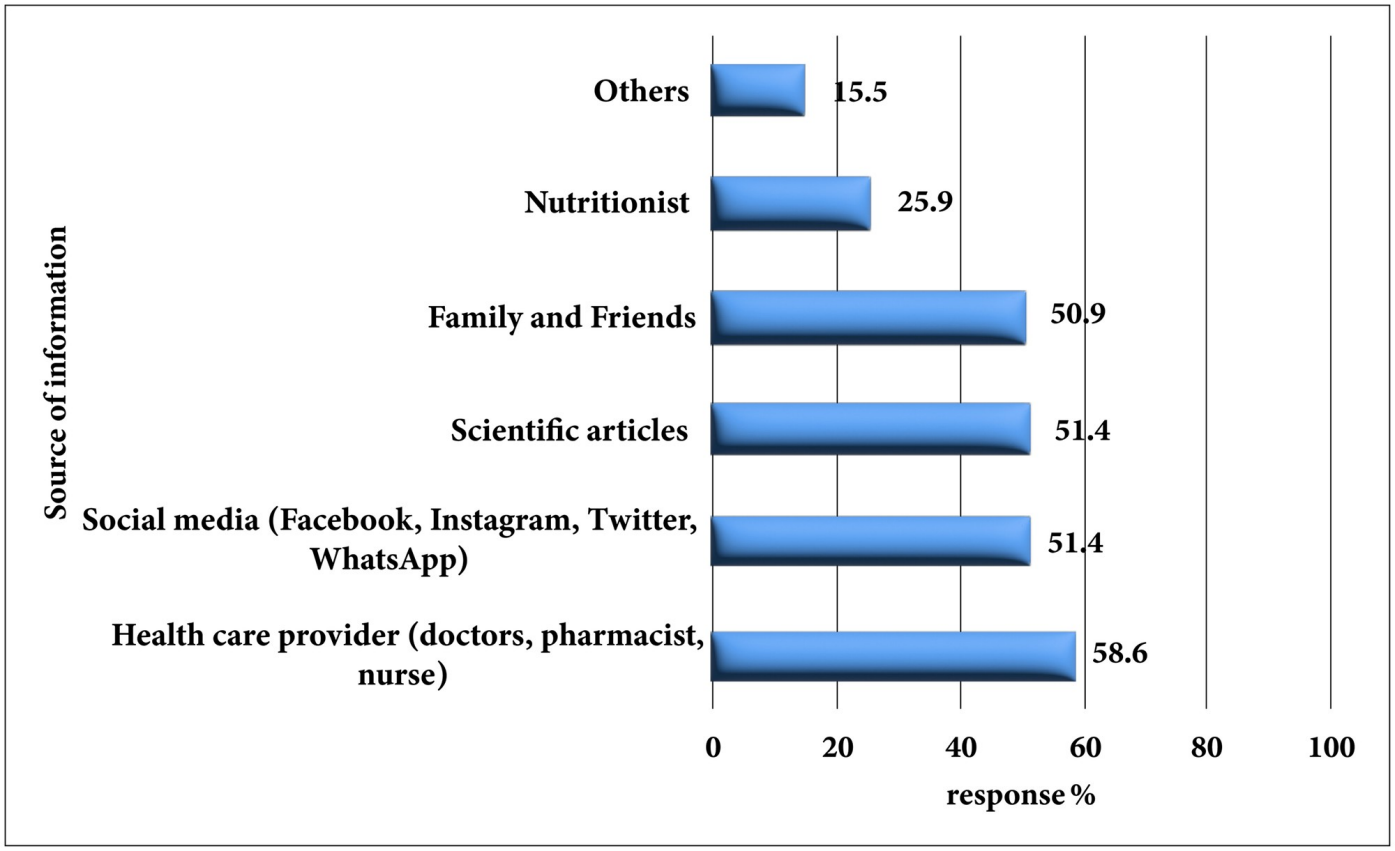
Source of information about antidiabetic medications uses for weight reduction (n = 220).

Furthermore, study participants declared that the main sources of information about these medications were healthcare providers (129, 58.6%), social media (113, 51.4%), scientific articles (113, 51.4%) and family/friends (112, 50.9%), Fig 2.

### Participants attitudes toward antidiabetic medications uses for weight reduction

Generally, the study participants demonstrated a neutral attitude toward antidiabetic medication use for weight reduction; the mean score was 3.35±0.45, as shown in Table 3. This indicates that although participants are aware of the medications, they do not hold strong opinions about their effectiveness or safety, reflecting overall uncertainty or indecision regarding their use. The majority of them agreed and strongly agreed that using these medications for weight loss should be under medical supervision (220, 56.7%). While most of the respondents strongly disagreed and disagreed with the rest of the attitude items in Table 3, including the presence of sufficient supervision in Jordan over these medication use (191, 49.1%).

**Table 3:** Participants’ attitude toward antidiabetic medications uses for weight reduction (n = 389).

Statements	n (%)				
	Strongly agree	Agree	Neutral	Disagree	Strongly disagree
• There is sufficient supervision in Jordan by the concerned authorities on the dispensing of antidiabetic medications used for weight loss purposes	17(4.37)	60(15.42)	121(31.11)	160(41.13)	31(7.97)
• These medications can be used for weight loss, but under medical supervision	70(17.99)	150(38.56)	124(31.88)	33(8.48)	12(3.08)
• The use of these medications is the first and best option for weight loss	10(2.57)	42(10.80)	112(28.79)	155(39.85)	70(17.99)
• These medications help you make the lifestyle changes you need to lose weight and improve your health	32(8.23)	95(24.42)	139(35.73)	94(24.16)	29(7.46)
• These medications are considered safe and can be used without complications	13(3.34)	52(13.37)	137(35.22)	138(35.48)	49(12.60)
• The effectiveness of these medications for weight loss is guaranteed	18(4.63)	73(18.77)	172(44.22)	86(22.11)	40(10.28)
• The efficacy of these medications is long-lasting	10(2.57)	41(10.54)	173(44.47)	125(32.13)	40(10.28)
**Attitude score** *	**Mean**	**SD**			
	**3.35**	**0.45**			

* Attitude Score (mean±SD) = 3.35±0.45. Attitude score was assessed using Likert scale with a maximum of 5 to a minimum of 1. Scores were categorized using Bloom’s cut-off points: < 3 (<59.%) were considered negative attitude, 3–3.9 (60.0–79.0%) as neutral and 4–5 (80.0–100.0%) as a positive attitude toward weight reduction medication.

### Participants practices toward antidiabetic medications uses for weight reduction

Among the study participants, only 48 (12.3%) of them have used medication for weight loss purposes in the last 12 months, Table 4. As most of them reported the use of (Glucophage^®^) (26, 54.2%) and (Ozempic^®^) (15, 31.3%). In addition, they declared the use of other methods for weight loss such as a healthy diet (42, 87.5%), exercising (40, 83.3%), use of other prescription medication (21, 43.8%) and use of herbals (20, 41.7%). More than three-quarters of these users utilized these medications from pharmacies (38, 79.2%) but reported difficulty obtaining them (36, 75.0%). The mean ±SD of body weight lost after the use of antidiabetic medication was 6.22 (±3.3) kg; however, 64.6% (n = 31) reported gaining weight after stopping these medications. Furthermore, 72.9% (n = 35) of the users experienced side effects from these medications, even 41.7% (n = 20) consulted a doctor before using medications.

**Table 4:** Participants’ practice toward antidiabetic medications uses for weight reduction.

Questions	n	%
**Have you used any medication for weight loss purposes in the last 12 months?** *		
No	341	87.7
**Yes**	**48**	**12.3**
**Which of the following antidiabetic medications have you used for weight loss purposes?** ^$^		
• (Ozempic^®^)	15	31.3
• (Saxenda^®^)	9	18.8
• (Mounjaro^®^)	3	6.3
• (Glucophage^®^)	26	54.2
**What other ways do you usually follow to lose weight in addition to using antidiabetic medications?** ^$^		
• Diet	42	87.5
• Other prescription medications	21	43.8
• Herbal products	20	41.7
• Exercising	40	83.3
• Others	9	18.8
**What is the source of these medications?** ^$^		
• Pharmacy	38	79.2
• private clinics/ medical centers	14	29.2
• Hospitals	13	27.1
• Facebook Pages	14	29.2
• Family and friends abroad	9	18.8
• Other sources	7	14.6
**Have you had difficulty obtaining these medications recently?** ^$^		
• Yes	36	75.0
• No	12	25.0
**If you have ever used these medications, how much weight (kg) did you lose?** (Mean±SD)	6.22±3.3 kg	
**Do you check the source of these medications when purchasing them?** ^$^		
• Yes	16	33.3
• No	32	66.7
**Did you consult a doctor before using these medications?** ^$^		
• Yes	20	41.7
• No	28	58.3
**Did you gain any weight back after you stopped using these medications?** ^$^		
• Yes	31	64.6
• No	17	35.4
**Have you experienced any side effects when using these medicines?** ^$^		
• Yes	35	72.9
• No	13	27.1
**Do you read medical information about the products you use to lose weight?** ^$^		
• Yes	41	85.4
• No	7	14.6

*Percentage was calculated based on total sample size (n = 389).

^**$**^Percentages were calculated based on the number of participants who have experience with weight reduction medications.

### Factors associated with knowledge and attitude toward antidiabetic medications uses for weight reduction

Table 5 demonstrates that many factors affected the participants’ knowledge and attitude scores significantly. Both scores were positively affected by female gender, high monthly income (>1000 JOD), medical profession and use of medication for weight loss in the last 12 months. In addition, the knowledge score was affected significantly by the presence of chronic diseases and increased BMI. In parallel, attitude score was affected positively by living in the center of Jordan and having medical insurance.

**Table 5:** Multiple linear regression for the factors affecting the knowledge and attitude scores among participants.

Factors	Knowledge score		Attitude score	
	Beta	*p-value*	Beta	*p-value*
• **Gender (Ref = female)**	0.191	**<0.001**	0.304	**<0.001**
• **Marital status (Ref = single)**	…….….…..	…….…..	-0.07	0.279
• **Place of residence (Ref = center)**	0.038	0.421	0.104	**0.042**
• **The monthly income of the family (Ref = >1000JD)**	0.176	**0.001**	0.171	**0.005**
• **Level of education (Ref = high school)**	…….….…..	…….…..	-0.042	0.476
• **Profession (Ref = non-medical)**	-0.367	**<0.001**	-0.139	**0.023**
• **Health insurance (Ref = Insured)**	0.013	0.793	0.097	**0.066**
• **Chronic diseases (Ref = No)**	-0.113	**0.025**	…….….…..	…….…..
• **Use of any medication for weight loss in the last 12 months (Ref = Yes)**	0.157	**0.001**	0.114	**0.03**
• **Height (cm)**	0.063	0.342	0.074	0.303
• **Weight (kg)**	0.124	**0.032**	0.057	0.366
• **BMI (Ref = Underweight)**	0.223	**0.002**	0.145	0.071
• **Age (in years)**	-0.142	0.051	…….….…..	…….…..
• **Attitude score**	-0.064	0.171	…….….…..	…….….…..
• **Knowledge score**	…….….…….	…….….…….	-0.063	0.2

*Significance measure at *p*-value<0.05 is presented in bold.

## Discussion

The aim of this study was evaluating Jordanian population knowledge, attitude and practices on the uses of antidiabetic medications such as GLP-1 receptor agonists *i*.*e*. (Ozempic^®^), ((Saxenda^®^), ((Mounjaro^®^) and biguanides such as (Glucophage^®^) for weight loss. The results of this study indicate fairly unconfident knowledge of the Jordanian population on the efficacy of selected antidiabetic agents in weight loss, however, it sheds the light on an alarmingly unsupervised uses of these medications to lose weight.

A recent systematic review reported an increase in the BMI by 11.1–72.4% in individuals > 16 years following COVID-19 lockdown during the period March to May 2020 [36]. Thus, this might have encouraged individuals to look for feasible methods to lose weight apart from traditional ones. Furthermore, the rising popularity in weight loss practices using medications that was introduced over different online platforms supported the off-label use of these medications to lose weight. For instance, previous studies of TikTok^®^ content analysis reported an alarmingly huge interest among platform users in watching videos under the hashtag (Ozempic^®^) with over 70 million views [37, 38]. This also accords with our observations, which showed that 51.4% (n = 113) of study participants’ knowledge about the use of antidiabetics for weight loss was from social media platforms.

A previous study that compared the prescription of anti-obesity medication before and after COVID-19 pandemic reported a 28.2% increase (n = 72/2250) in prescribing of Liraglutide (*i*.*e*. (Saxenda^®^) [39]. Comparably, 18.8% (n = 9/48) of our study participants who used antidiabetics for weight loss used (Saxenda^®^) and most probably without medical advice as indicated by 58.3% (n = 28/48) of antidiabetics users for weight loss purposes. This could be attributed to the possibility of self-medication that is quite prevalent in Jordan [40].

Metformin that was first licensed under the trade name (Glucophage^®^), and is the first line to treat type II diabetes, demonstrated a profound effect in weight loss, yet, not FDA approved for this purpose [41]. Massive literature is available about the beneficial impact of metformin in weight reduction, especially, in people with insulin resistance and Jordanian population [6, 16, 42, 43]. In this study, we provide enhanced insights into the efficacy of metformin for weight loss within the Jordanian population, which has one of the highest rates of diabetes, where many individuals may have experienced this firsthand. For instance, a previous study have reported a mean loss of 5.8±7.0 kg when metformin was administered to non-diabetic patients with a BMI≥ 27 kg/m^2^ [42]. This decrease is comparable to the mean± SD weight loss reported in our study 6.22 (±3.3) kg. However, the overall knowledge score was considered poor (6.6±3.03) which might be due to people relying on different sources for medical information other than healthcare providers such as social media (51.4%, n = 113) and family and friends (50.9%, n = 112) [44]. Nevertheless, this might impose certain degree of health risk due to the absence of regulatory authorities’ supervision of items sold over non-medical platforms *(e*.*g*. Facebook pages). In addition, 66.7% (n = 32/48) of these medications’ users do not check the source of their medications [45]. Moreover, relying on web-based medical information can pose health risks, particularly when it leads users to delay necessary treatment, ultimately impacting their overall health. A recent study highlighted various potential consequences of misinformation, including physical harm, emotional distress, financial loss, and the creation of false hope [46].

A previous mail-push-to-web survey that was conducted in the United States to assess population knowledge of the FDA supervision to both prescription medications approvals and advertising, found that 31% (n = 1744) think that the FDA approved medications advertisements [47]. On the contrary, our study participants generally, demonstrated good knowledge about FDA approvals for the selected antidiabetic agents commonly utilized for weight loss. The highest score was for (Mounjaro^®^) with a knowledge score of 85.91% (n = 143) [47]. However, this study supports the evidence from previous observations indicating regulatory agencies supervision as insufficient [47]. On the other hand, the participants of our study demonstrated fairly superior knowledge about the use of (Ozempic^®^) and (Saxenda^®^) in weight loss (47.27% (n = 104), 44.55% (n = 98), respectively) compared to another study conducted on Saudi population (33%, 31.8%, respectively) [48]. Overall, humble knowledge was demonstrated in both studies regardless of the high prevalence of obesity. This might be attributed to the fact that sources of information about these medications are probably from individuals of non-medical background such as social media platforms bloggers (51.4%, n = 113). Albeit pharmacies are the main source for obtaining weight loss medications, a 29.2% (n = 14/48) of study participants who used antidiabetics for weight loss declared that they purchase these medications over Facebook^®^ pages and 14.6% from other unrecognized sources. This might impose a huge risk to consumers’ health as previous warnings were issued about counterfeited Semaglutide that was replaced with insulin [49].

Female gender was one of the factors affecting knowledge and attitude scores (*p*< 0.001). These results support previous research findings by *Tsai et*. *al* that reported poor weight loss perception OR = 0.36) and attempted weight loss (OR = 0.39) by men compared to women [50]. Additionally, 3.1% of women have reported willingness to use prescription pills to lose weight compared to 0.5% in men [50]. Similarly, in this study, (n = 38/48), 79.16% of antidiabetic medication users for weight loss were female. In agreement with *Almubarak et*. *al* findings, men showed poor attitude compared to women when it comes to weight loss strategies [51]. This could be explained by the gender-related aesthetic interest, primarily due to the endorsement of the fit shape [52]. Furthermore, dieting (87.5%, n = 42) and exercising (83.3%, n = 40) were the most common practices to lose weight in conjunction to medications which is comparable to a report published about Saudi female attitudes and practices in weight management [53].

As anticipated, the presence of a chronic disease was a significant factor affecting knowledge regarding the use of anti-diabetic medications for weight loss (*p* = 0.025). A plausible explanation for this finding is that these patients may have used these medications for managing their condition and subsequently experienced weight loss as an unintended side effect [54]. In addition, using antidiabetic agent in the last 12 months to lose weight was a significant factor affecting both knowledge and attitude (*p* = 0.001, *p* = 0.03, respectively). This intriguing finding could be attributed to the possibility that study participants using antidiabetic medications for weight loss may have experienced both weight reduction and actively searched online databases for medical information. However, 58.3% (n = 28) declared that they do not consult their doctors before utilizing these medications to lose weight. This practice is alarming due to the possibility of administering these medications in presence of precautions and/or contraindications of use.

Regarding BMI, We found that BMI significantly predicts the knowledge about antidiabetic medications in weight loss practices (p = 0.002). This could be explained by the fact that people with high BMI (*i*.*e*. indicating overweight or obesity) are more keen to utilize different methods to lose weight and consequently more educated about the off-label uses of these medications [55, 56].

In general, participants’ attitudes, both who used or did not use these medications, was neutral. This could be a result of the limited number of medication users among the study sample (*i*.*e*. 12.3%, n = 48/389). Despite the rigorous supervision by regulatory authorities in Jordan, respondents had a negative attitude towards the level of supervision about these medications (191, 49.1%, n = 191). This could be ascribed to the advertisement that blew the social media platforms about using these medications for weight loss, especially by celebrities and bloggers [37]. Hence the high percentage of respondents who obtained these medications from sources other than legalized suppliers *i*.*e*. pharmacies, (36, 75.0%). These findings offer support for the concept that regulating the dispensing of medications for off-label use is essential to ensure the safety and adequacy of the national drug supply. Especially, there are worldwide press reports about shortages in GLP-1 receptor agonists [57]. This is in line with our findings, as 75% of study Participants who used antidiabetics for weight loss (n = 36) reported difficulty in obtaining (Ozempic^®^), (Saxenda^®^), (Mounjaro^®^). Thus, relying on other sources to acquire these medications such as Facebook^®^ pages and other unidentified sources (14.6%, n = 7). As a result and due to the social media propaganda, unofficial suppliers started to either smuggle these products from other countries or to fake preparations such as (Ozempic^®^) and (Saxenda^®^) by adding insulin instead [49]. This is rather difficult to supervise by regulatory authorities, especially, if the quantity being smuggled is small. Thus, it is highly recommended to educate the public about the harm of receiving these medications without regulatory supervision which might place them at risk of developing severe adverse effects. However, more research on this topic needs to be undertaken before establishing an association between medications smuggling and its influence on consumers’ health. Moreover, further studies are warranted to investigate the influence of social media on consumers’ behavior in purchasing medications in general with a special focus on weight loss medications.

Despite the insights and findings of this study about Jordanian population use of antidiabetics for weight loss, further investigations are required to identify what other prescription medications they use to lose weight. Particularly, 43.8% (n = 21) of users reported using medications other than (Ozempic^®^), (Saxenda^®^), (Mounjaro^®^) and (Glucophage^®^). Furthermore, assessing the long-term impact of using antidiabetics for weight loss purposes. Also, a global assessment for the usage GLP-1 receptor agonists specifically for weight loss is highly required, especially, there is a huge social media debate about it.

Limitations to this study include that most of respondents were from central of Jordan who might have better access to the internet compared to other areas as the questionnaire was distributed online. Additionally, the self-reported nature of the study which might have imposed some bias to the results. However, this was not presumably influencing the results as the knowledge score was fair.

## Conclusion

The objective of the current study was to assess knowledge, attitude and practices of Jordanian population towards using antidiabetic agents for the purposes of weight loss. The most obvious findings included that gender, BMI, health profession, monthly income, chronic diseases and the use of these medications were factors affecting Jordanian population knowledge of antidiabetics for weight loss. Also, the percentage of (Ozempic^®^) and (Glucophage^®^) users for weight loss purposes is considered high besides a substantial percentage obtaining these medications from social media retailers or other undeclared sources. Taken together, these results warrant the need to educate the public about the risks of purchasing medications and specifically, those being marketed for weight loss purposes from unauthorized resources due to authenticity issues which might affect their overall health and desired outcome. In addition to initiate stringent act by regulatory authorities on prescription and dispensing of these medications for weight loss purposes.

## Supporting information

S1 Appendix The questionnaire. (DOCX)
