# Supplementary material for: Knowledge, attitudes, and practices towards the use of GLP-1 receptor agonists for weight loss among the general population in Jordan; A cross-sectional study
Source: PLoS One. 2024 Dec 5;19(12):e0314407. doi: 10.1371/journal.pone.0314407 (PMC11620468; doi:10.1371/journal.pone.0314407)
Supplement: S1 Appendix — (DOCX) [file pone.0314407.s001.docx]

**Assessment of the knowledge, awareness and practices of the Jordanian people on the uses of diabetes treatments for weight loss purposes**

1. Your participation in filling out this questionnaire is appreciated and will be treated confidentially. Would you like to participate?

- Yes
- No
- Section Two (Personal Information)

2. Gender

- Male
- Female

3. Height (cm)

.................................................. .

4. Weight (kg)

.................................................. .

5. Age (in years)

.................................................. .

6. Marital status

- Bachelor
- married
- Other

7. Place of residence

- North governorates (Irbid, Jerash, Ajloun, Mafraq)
- Central governorates (Amman, Zarqa, Balqa, Madaba)
- Southern governorates (Karak, Tafila, Ma'an, Aqaba)

8. The monthly income of the family

- Less than 300 dinars
- 300-700 dinars
- 701-1000 dinars
- More than 1000 dinars

9. The highest degree or level of education you have completed:

- High School
- Vocational education
- Diploma
- Bachelor's degree
- Postgraduate degree (Master's, Ph.D.)

10. Occupation (profession)

- Medical occupations (medicine, pharmacy, nursing, laboratories...etc.)
- Non-medical occupations (engineering, information technology, arts, Craft occupations, etc.)
- Not applicable

11. Do you have health insurance?

- Yes
- no

12. Do you suffer from chronic diseases?

- Yes
- no

13. If yes, what are these diseases?

Diabetes:

- yes
- no

High blood pressure:

- Yes
- No

Cardiovascular disease:

- Yes
- no

Thyroid diseases:

- Yes
- no

Hyperlipidemia (High Cholesterol):

- Yes
- no

Obesity:

- yes
- no

Others:

- yes
- no
- Section three ((a) Jordanian public knowledge of antidiabetic medications)

14. As far as you know, is it possible to use some antidiabetic medications to lose weight?

- Yes
- no
- not sure
- Section four ((b) Jordanian public knowledge of antidiabetic medications)

15. To the best of your knowledge, which of the following medications can be used to lose weight?

Ozempic:

- Yes
- No
- I don't know

Saxenda:

- Yes
- No
- I don't know

Mounjaro:

- Yes
- No
- I don't know

Glucophage:

- Yes
- No
- I don't know

Insulin:

- Yes
- No
- I don't know

Amaryl:

- Yes
- No
- I don't know

Jardiance:

- Yes
- No
- I don't know

16. Which of the following medications is approved to be used for weight loss by the regulatory authorities such as the Food and Drug Administration?

Ozempic:

- Yes
- No
- I don't know

Saxenda:

- Yes
- No
- I don't know

Mounjaro:

- Yes
- No
- I don't know

Glucophage:

- Yes
- No
- I don't know

Insulin:

- Yes
- No
- I don't know

Amaryl:

- Yes
- No
- I don't know

Jardiance:

- Yes
- No
- I don't know

17. To the best of your knowledge, what are the side effects of these medications?

Nausea and vomiting:

- Yes
- No
- I don't know

Diarrhoea:

- Yes
- No
- I don't know

Abdominal pain:

- Yes
- No
- I don't know

Headache:

- Yes
- No
- I don't know

Fatigue:

- Yes
- No
- I don't know

Weight gain:

- Yes
- No
- I don't know

Skin swelling or irritation where the needle was inserted:

- Yes
- No
- I don't know

Dark patches of skin:

- Yes
- No
- I don't know

Unwanted sexual responses (sexual dysfunction or arousal):

- Yes
- No
- I don't know

Depression:

- Yes
- No
- I don't know

Pancreatitis:

- Yes
- No
- I don't know

Tumours such as thyroid tumour:

- Yes
- No
- I do not know

Urinary tract infection:

- Yes
- No
- I don't know

Others:

- Yes
- No
- I don't know

18. What is the source of your information about the effectiveness of these medications in weight loss?

Health care provider (doctor, pharmacist, nurse):

- Yes
- No

Nutritionist:

- Yes
- No

Social media (Facebook, Instagram, Twitter, WhatsApp):

- Yes
- No

Scientific articles:

- yes
- no

Family and friends

- Yes
- no

Others:

- yes
- no
- Section Five (Assessment of Jordanian society's awareness of the uses of antidiabetic medications for weight loss)

19. To what extent do you agree with the following statements:

There is sufficient supervision in Jordan by the concerned authorities on the dispensing of antidiabetic medications used for weight loss purposes:

- Strongly Agree
- Agree
- Neutral
- Disagree
- Strongly disagree

These medications can be used for weight loss, but under medical supervision:

- Strongly Agree
- Agree
- Neutral
- Disagree
- Strongly disagree

The use of these medications is the first and best option for weight loss:

- Strongly Agree
- Agree
- Neutral
- Disagree
- Strongly disagree

These medications help you make the lifestyle changes you need to lose weight and improve your health:

- Strongly Agree
- Agree
- Neutral
- Disagree
- Strongly disagree

These medications are considered safe and can be used without complications:

- Strongly Agree
- Agree
- Neutral
- Disagree
- Strongly disagree

The effectiveness of these medications for weight loss is guaranteed:

- Strongly Agree
- Agree
- Neutral
- Disagree
- Strongly disagree

The efficacy of these medications is long lasting:

- Strongly Agree
- Agree
- Neutral
- Disagree
- Strongly disagree
- Section Six (Jordanian population practices regarding the use of injectable antidiabetic medications for weight loss purposes)

20. Have you used any medication for weight loss purposes in the last 12 months?

- Yes
- no
- Section Seven (Jordanian population practices regarding the use of antidiabetic medications for weight loss purposes)

21. Which of the following antidiabetic medications have you used for weight loss purposes?

Ozempic:

- Yes
- No

Saxenda:

- Yes
- No

Mounjaro:

- Yes
- No

Glucophage:

- Yes
- No

Insulin:

- Yes
- No

Amaryl:

- Yes
- No

Jardiance:

- Yes
- No

22. What other ways do you usually follow to lose weight in addition to using antidiabetic medications?

Diet:

- Yes
- no

Other prescription medications:

- Yes
- No

Herbal preparations:

- Yes
- no

Exercising:

- Yes
- no

Others:

- Yes
- no

23. Where do you get these medicines from?

Pharmacy:

- Yes
- no

private clinics/ medical centers:

- Yes
- no

Hospitals:

- Yes
- no

Facebook Pages:

- Yes
- no

Family and friends abroad:

- Yes
- no

Other sources:

- Yes
- no

24. Have you had difficulty obtaining these medications recently?

- Yes
- no

25. If you have ever used these medications, how much weight (kg) did you drop?

.................................................. ....................

26. Do you check the source of these medications when purchasing them?

- Yes
- no

27. How do you verify the source of these medications?

JPA pricing label

- Yes
- no

Barcode (serial number)

- Yes
- no

Manufacturers’ name

- Yes
- no

Refer to the pharmacist

- Yes
- no

Others

- Yes
- no

28. Did you consult a doctor before using these medications?

- Yes
- no

29. Did you gain any weight back after you stopped using these medications?

- Yes
- no

30. Have you experienced any side effects when using these medicines?

- Yes
- no

31. Do you read medical information about the products you use to lose weight?

- Yes
- no

32. If you would like to share any additional information regarding the use of antidiabetic medications for weight loss, please write it in the box below.

.................................................. .................................................. ..............
